# Editorial: Cognition, Behavior and Cybersecurity

**DOI:** 10.3389/fpsyg.2021.728132

**Published:** 2021-08-05

**Authors:** Paul Watters, Nalin Asanka Gamagedara Arachchilage, David Maimon, Richard Keith Wortley

**Affiliations:** ^1^Department of Security Studies and Criminology, Macquarie University, Sydney, NSW, Australia; ^2^Department of Computer Science and Information Technology, La Trobe University, Melbourne, VIC, Australia; ^3^Department of Criminal Justice and Criminology, Georgia State University, Atlanta, GA, United States; ^4^Department of Security and Crime Science, University College London, London, United Kingdom

**Keywords:** cybersecurity, cognition, behaviour, malware, phishing

Cybersecurity appears to be the ultimate paradox: while cybersecurity budgets are increased every year, and a vast array of new security products and services appear in the market, cyber attacks have been increasing in scale and scope every year. 2020 will perhaps be remembered as the “Year of Ransomware” as malware authors rendered useless every technical attempt to block them from attacking critical systems and data.

In this Research Topic, we have tried to present an alternative but highly complementary view to the almost total focus on purely technical solutions in cybersecurity, namely—that cybersecurity attacks ultimately succeed because they target the cognitive and behavioural vulnerabilities of ordinary users, and that for attacks to be prevented (at best) or mitigated (at least), user-focused techniques must be researched, fostered, and developed.

The small but growing band of dedicated researchers and practitioners in human factors in cybersecurity is making real inroads into developing a holistic view on how fundamental psychological principles—cognition, behaviour, perception, motivation, and emotion, to name but a few—can be readily understood within a sociotechnical context to be the primary basis for embracing a security-by-design philosophy.

Humans are complex beasts. They are motivated by a range of conscious factors and unconscious biases to make decisions that are highly exploitable by cybercriminals. Phishing texts, for example, are carefully designed to create a sense of urgency in the receiver, while malware delivery relies on the routinised habit of clicking on links. More generally, scammers exploit our inability to reconcile conflicting information in time-pressured circumstances, and our susceptibility to buy overpriced commodities during a market bubble as described in greater fool theory.

If there is one conclusion that we can draw from the body of work presented in this Research Topic, it is that computer scientists, psychologists, designers, and policy makers need to work much more closely together, to create the policy settings, technical solutions, and user validation for the secure apps and trustworthy infrastructure of tomorrow. On the one hand, a very narrow and perhaps technologists' view of user behaviour lacks sophistication, and designs ignoring psychological views are prone to exploitation.

On the other hand, more behaviourally-focused cybersecurity controls (such as auditing) can lead to abstractions (such as checklisting) that often lack the empirical connection to a deep understanding of how technologies actually work. The policy settings within which systems are allowed to be developed and operated need serious attention: Europe's General Data Protection Regulation (GDPR) speaks of “Privacy By Design,” and Australia's Privacy Act (1988) relies on organisations taking “reasonable steps” to protect personal data, but there are few concrete pathways or examples of how this may be achieved using psychologically valid principles. Further integration, engagement, and mutual understanding is necessary to improve system design, and ultimately, better social and commercial outcomes.

## Author Contributions

All authors listed have made a substantial, direct and intellectual contribution to the work, and approved it for publication.

## Conflict of Interest

The authors declare that the research was conducted in the absence of any commercial or financial relationships that could be construed as a potential conflict of interest.

## Publisher's Note

All claims expressed in this article are solely those of the authors and do not necessarily represent those of their affiliated organizations, or those of the publisher, the editors and the reviewers. Any product that may be evaluated in this article, or claim that may be made by its manufacturer, is not guaranteed or endorsed by the publisher.

